# FACTORS ASSOCIATED WITH THE PSYCHOLOGICAL WELL-BEING OF OLDER MEN WHO HAVE SEX WITH MEN IN THE CHINESE POPULATION

**DOI:** 10.1093/geroni/igad104.2167

**Published:** 2023-12-21

**Authors:** Alex Siu Wing Chan, Elsie Yan

**Affiliations:** The Hong Kong Polytechnic University, Hong Kong, Hong Kong; THe Hong Kong Polytechnic University, Hong Kong, Hong Kong

## Abstract

Older men who have sex with men (OMSM) experience discrimination based on their age and sexual orientation, which presents major challenges to their health. Existing studies have examined discrimination experienced by younger MSM. This study aims to examine the discrimination experienced by OMSM in the People’s Republic of China (PRC), Hong Kong, and Taiwan. The eligible participants for this research comprised OMSM aged over 60 years. The demographic information of the participants was collected. Particularly, the General Health Questionnaire-12 (GHQ-12), Personal Social Capital Scale (PSCS), Perceived Acceptance Scale (PAS), and Global Index on Legal Recognition of Homosexual Orientation (GIRLHO) scales were used to assess psychological well-being, discrimination, social capital, perceived acceptance, and legal inclusion. Discrimination differs across PRC, Hong Kong, and Taiwan (F(2, 450) = 112.07, p < .001), and it also has a negative impact on psychological well-being (B = -1.16, p <.001). Social capital was found to have a negative impact on psychological well-being (B = -3.38, p < 0.001), and a positive impact on legal inclusion (B = 1.55, p< 0.001). Lastly, social acceptance has a positive impact on psychological well-being (B = 0.52, p = .035). This study provides new insights into the negative impact of discrimination on the psychological health of OMSM and mitigating factors representing the positive effects. However, future studies are necessary to bridge the literature gap regarding the associations between the different factors described in this study.

